# Metabolic Pathways and Molecular Regulatory Mechanisms of Fruit Color Change During Greening Stage of Peppers (*Capsicum annuum* L.)

**DOI:** 10.3390/ijms26104508

**Published:** 2025-05-09

**Authors:** Mengyuan Wei, Junqin Wen, Yanjing Ren, Dengkui Shao, Yayi Wang, Jiang Li, Quanhui Li

**Affiliations:** 1 Academy of Agriculture and Forestry Sciences, Qinghai University, Xining 810016, China; monyan_w@163.com (M.W.); 2021990055@qhu.edu.cn (J.W.); 2018990082@qhu.edu.cn (Y.R.); 2006990015@qhu.edu.cn (D.S.); 2010990020@qhu.edu.cn (Y.W.); 2004990019@qhu.edu.cn (J.L.); 2 Laboratory for Research and Utilization of Qinghai Tibet Plateau Germplasm Resources, Xining 810016, China; 3 Key Laboratory of Germplasm Resources Protection and Genetic Improvement of the Qinghai-Tibet Plateau in Ministry of Agriculture and Rural, Xining 810016, China

**Keywords:** *Capsicum annuum* L., flavonoid metabolism, chlorophyll metabolism, metabolomics, transcriptomics, fruit color

## Abstract

Our multi-omics investigation of pepper fruit coloration dynamics demonstrates that the coordinated regulation of flavonoid accumulation and chlorophyll retention underpins the distinct pigmentation patterns between dark green (XHB) and light green (QL2017) cultivars. Through the integrated analysis of three developmental stages (10–30 DPA), we identified 989 differentially accumulated metabolites (DAMs) and 810 differentially expressed genes (DEGs), with flavonoid biosynthesis, phenylpropanoid metabolism, and chlorophyll turnover pathways pinpointed as central regulatory hubs. Notably, key metabolites such as quercitrin, kaempferol-3-O-rhamnoside, and cinnamic acid were significantly enriched in dark green fruits (XHB), coinciding with enhanced antioxidant activity and delayed chlorophyll degradation. Transcriptomic data revealed the coordinated upregulation of chlorophyll biosynthesis genes (*COX15*, *POR*) and light-harvesting complex components (*Lhcb1*, *Lhcb2*), while *PAO*—a pivotal chlorophyll catabolism gene—also exhibited elevated expression. Co-expression network analysis highlighted *scopoletin GTase*, *F5H*, *CCR*, and *CAD* as hub genes regulating flavonoid biosynthesis. qRT-PCR validation confirmed high consistency with transcriptomic trends (*r* > 0.85, *p* < 0.01). Our findings propose a synergistic model wherein flavonoid accumulation and chlorophyll metabolic dynamics jointly orchestrate green fruit pigmentation, offering novel insights and molecular targets for the precision breeding of pepper fruit coloration.

## 1. Introduction

The genus *Capsicum* (*Capsicum annuum* L.), native to tropical regions of Latin America, ranks among the earliest domesticated vegetable crops with global cultivation distribution [1]. Fruit coloration constitutes a key visual quality parameter that directly influences market selection and breeding strategies. By utilizing genetic resources and inducing mutagenesis, researchers have developed pepper varieties exhibiting distinct pericarp pigmentation patterns, effectively meeting diversified market demands [2]. Modern breeding innovations now produce peppers displaying unconventional hues like white, purple, and black, complementing traditional red, orange, and yellow varieties [3]. Current research confirms that pepper pigmentation primarily results from dynamic accumulation patterns of chlorophylls, flavonoids, and carotenoids in fruit tissues, with the former two pigments predominantly determining immature fruit coloration [4,5].

Chlorophyll metabolism plays a crucial role in determining the skin color of plant fruits. Key genes involved in chlorophyll biosynthesis have been identified in the leaves and fruits of numerous plant species [6,7]. Chlorophyll, the quintessential photosynthetic pigment, is comprised of two distinct molecular species: chlorophyll a and chlorophyll b. These ubiquitous plant pigments confer the characteristic green pigmentation observed in the pericarp [8]. Chlorophyll (Chl) biosynthesis in plants unfolds through three sequential phases, with the initial two phases overlapping tetrapyrrole synthesis pathways common to photosensitive pigments [9,10]. The process begins with L-glutamate activation by glutamyl-tRNA synthetase (GluRS), followed by its reduction to δ-aminolevulinic acid (ALA) via GluTR and GSA-AM, coordinated within an enzyme complex to optimize substrate utilization [11]. In phase two, ALA is converted to protoporphyrin IX (Proto IX) through intermediates such as porphobilinogen and coproporphyrinogen III, driven by enzymes including PBGD and CPOX [10,12]. The final phase, unique to Chl, initiates with magnesium chelatase (MgCh) inserting Mg^2+^ into Proto IX. Subsequent steps involving POR and CHLG yield chlorophyll a, which is oxidized by CAO to form chlorophyll b, marking the pathway’s divergence from other tetrapyrroles [13,14,15]. The biosynthesis of chlorophyll involves stage-specific regulation by enzymatic components (e.g., POR, GLK, PAO) and environmental modulators including light intensity and ionic stressors. While transcriptomic analyses have identified core regulatory networks, the precise coordination between genetic determinants and biotic/abiotic factors remains incompletely characterized, necessitating comprehensive investigations into their crosstalk mechanisms [8,16,17,18,19].

Flavonoids, secondary metabolites derived from the phenylalanine pathway, are ubiquitously distributed across plant tissues and fulfill critical physiological roles, including organ pigmentation, UV radiation protection, pathogen defense, and antioxidant activity [20,21,22,23]. Within the phenylpropanoid biosynthetic pathway, p-coumaroyl-CoA, synthesized from cinnamic acid, serves as the substrate for chalcone synthase (CHS), catalyzing the formation of naringenin chalcone. Subsequent stereospecific cyclization by chalcone isomerase (CHI) yields naringenin, a pivotal intermediate in flavonoid metabolism. Naringenin undergoes further enzymatic modifications mediated by key structural enzymes, including flavanone 3-hydroxylase (F3H), flavonol synthase (FLS), and anthocyanidin synthase (ANS), which collectively orchestrate the biosynthesis of diverse secondary metabolites such as flavonols, anthocyanins, proanthocyanidins, flavones, and isoflavones. These compounds are essential for both pigmentation and stress responses in plant systems [24,25,26].

The integration of metabolomic and transcriptomic profiling has emerged as a powerful approach for elucidating complex biosynthetic networks in chili pepper research. Existing studies have successfully applied these omics tools to investigate volatile compound biosynthesis [27,28], fruit color transition dynamics [29,30,31], thermotolerance mechanisms [32], and plant–pathogen interactions [33]. While such investigations have advanced our understanding of Capsicum biology, the specific molecular interplay governing light-to-dark green pigmentation shifts in immature fruits remains underexplored through combined metabolite–gene network analysis.

In the present investigation, we employed ‘XHB’, a cultivar characterized by dark green fruit coloration, alongside its light green mutant counterpart ‘QL2017’. Through integrated transcriptomic and metabolomic analysis, we have delineated the pivotal roles of flavonoid, phenylpropanoid, and chlorophyll metabolic pathways in fruit color formation. Furthermore, we have identified key regulatory genes involved in the modulation of fruit color transition, thereby providing novel insights into the molecular regulatory mechanisms underlying fruit coloration during the green ripening phase of pepper development.

## 2. Results

### 2.1. Metabolite Analysis Results

The comprehensive metabolite profiling revealed distinct biochemical signatures between dark green (DG) and light green (LG) pepper fruits across developmental stages. We identified 989 metabolites spanning 11 principal classes, with flavonoids emerging as the most abundant category (211 metabolites), followed by phenolic acids (139) and alkaloids (122).

**Figure 1:**
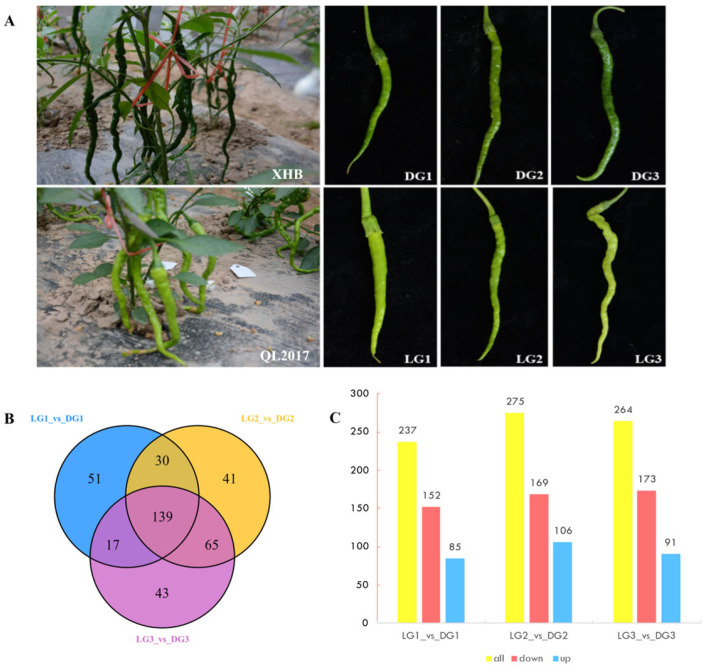
Comparative analysis of DAMs between DG and LG pepper fruits at three developmental stages. (**A**) Phenotypic comparison of DG and LG fruits at 10 (D1), 20 (D2), and 30 (D3) days post-anthesis. (**B**) Venn diagram illustrating the distribution of DAMs across developmental stages. (**C**) The number of up-regulated and down-regulated DAMs at each developmental stage.

Differentially accumulated metabolites (DAMs) between DG and LG were analyzed at three developmental stages: 10 days (d), 20 d, and 30 d post-anthesis (Figure 1). At 10 d post-anthesis (LG1 vs. DG1), 237 DAMs were identified, comprising 152 down-regulated, 85 up-regulated, and 51 unique metabolites. At 20 d post-anthesis (LG2 vs. DG2), 275 DAMs were identified, including 169 down-regulated, 106 up-regulated, and 41 unique metabolites. At 30 d post-anthesis (LG3 vs. DG3), 264 DAMs were identified, consisting of 173 down-regulated, 91 up-regulated, and 43 unique metabolites. Analyses of differential metabolites across the three phases revealed a progressive rise in downregulated compound quantities.

**Figure 2:**
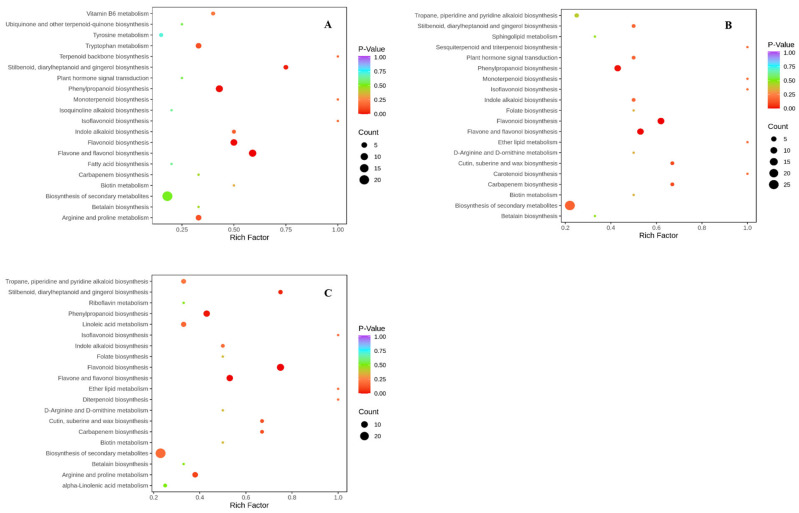
KEGG pathway enrichment analysis of differentially expressed metabolites between DG and LG pepper fruits at three developmental stages. (**A**) LG1 vs. DG1, (**B**) LG2 vs. DG2, (**C**) LG3 vs. DG3. The *x*-axis represents the Rich Factor for each pathway; the *y*-axis shows pathway names. Point color intensity corresponds to the −log_10_(*p*-value), with deeper red indicating greater significance. Point size reflects the number of enriched differentially expressed metabolites per pathway.

Comparative KEGG pathway analyses of DG and LG pepper fruits across three developmental stages (10, 20, 30 DPA) revealed conserved enrichment patterns in phenylpropanoid/flavonoid metabolism (Figure 2). Additionally, the biosynthesis pathways of stilbenoids, diarylheptanoids, and gingerols were significantly enriched at 10 d and 30 d post-anthesis. Furthermore, the biosynthesis of the secondary metabolites pathway exhibited the highest number of differentially enriched metabolites across all three stages.

### 2.2. Transcriptome Analysis Results

The raw RNA-Seq data were preprocessed to obtain high-quality clean reads (Supplementary Table S2), with an average GC content of 41.98% and a Q30 score of 93.02%. These metrics confirm that the sequencing quality meets established standards and is suitable for downstream analyses. The clean reads were aligned to the reference genome, achieving an average mapping rate of 94.18%. This high mapping rate indicates precise assembly of the reference genome, species consistency, and the absence of contamination in the experimental samples.

**Figure 3:**
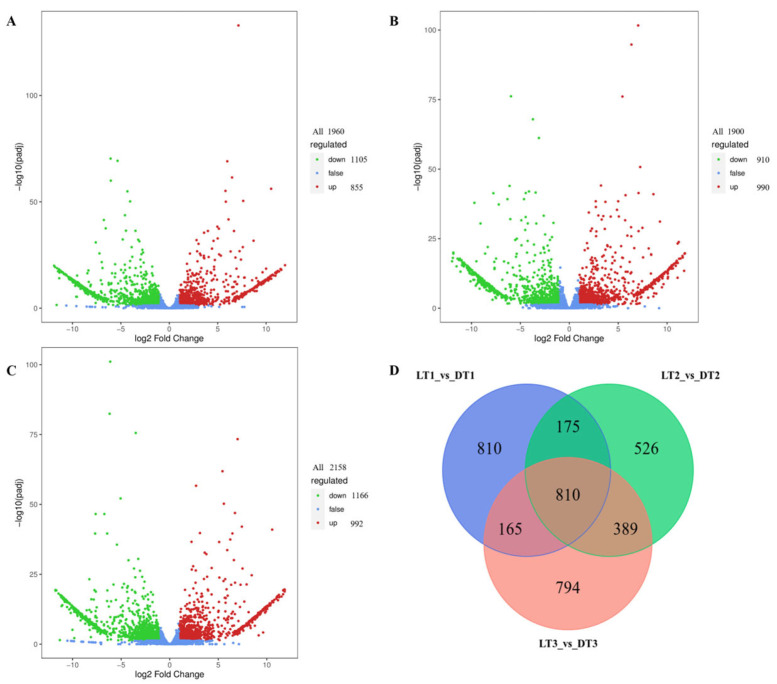
Differential gene expression analysis. (**A**–**C**) Volcano plots of DEGs for LG1 vs. DG1, LG2 vs. DG2, and LG3 vs. DG3. The *x*-axis represents log_2_ fold change in gene expression, and the *y*-axis represents the −log_10_(*p*-value). Red, green, and blue points denote up-regulated, down-regulated, and non-significant genes, respectively. (**D**) Venn diagram illustrating the overlap of DEGs across the three developmental stages.

Using stringent screening criteria (|log_2_Fold Change| ≥ 1 and FDR < 0.05), 810 differentially expressed genes (DEGs) were identified as common across all three developmental stages (Figure 3). At 10 days after anthesis (LG1 vs. DG1), there were 1960 differentially expressed genes, of which 855 were upregulated, 1105 were downregulated, and 810 were unique differentially expressed genes. At 20 days after anthesis (LG2 vs. DG2), there were 1900 differentially expressed genes, of which 990 were up-regulated, 910 were down-regulated, and 526 were unique differentially expressed genes. At 30 days after anthesis (LG3 vs. DG3), there were 2158 differentially expressed genes, of which 992 were up-regulated, 1166 were down-regulated, and 794 were unique differentially expressed genes.

**Figure 4:**
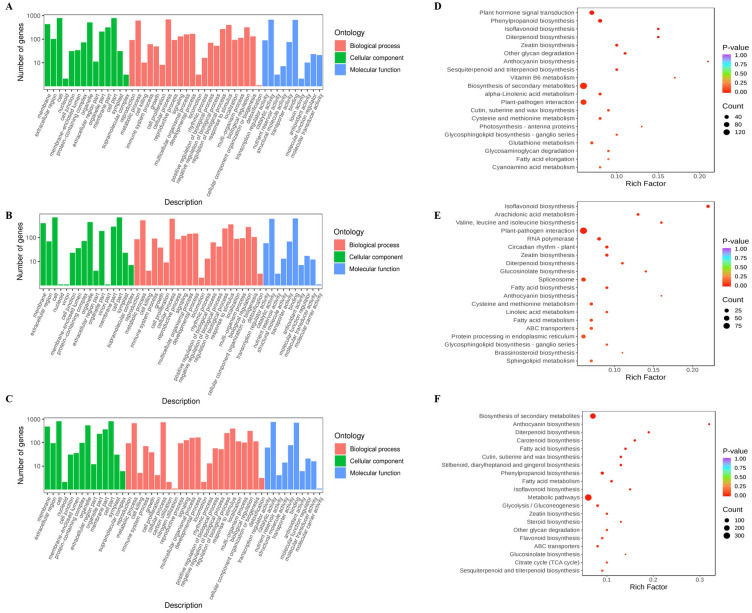
GO and KEGG enrichment analyses of DEGs. (**A**–**C**) GO classification histograms for LG1 vs. DG1, LG2 vs. DG2, and LG3 vs. DG3. The *x*-axis represents secondary GO terms, while the *y*-axis indicates the number of DEGs annotated to each term. (**D**–**F**) KEGG pathway enrichment profiles for LG1 vs. DG1, LG2 vs. DG2, and LG3 vs. DG3. The *x*-axis represents the Rich Factor, the *y*-axis shows pathway names, and the color intensity of the dots corresponds to the −log_10_(*p*-value), with deeper red indicating higher significance. Dot size reflects the number of DEGs enriched in each pathway.

Gene Ontology (GO) enrichment analysis was performed on the DEGs, revealing annotations across three major categories: Molecular Function, Biological Process, and Cellular Component (Figure 4A–C). Within the Cellular Component category, the most significantly enriched terms included Cell, Cell Part, and Organelle. In the Biological Process category, the most enriched terms were Metabolic Process, Cellular Process, Response to Stimulus, and Regulation of Biological Process. Within the Molecular Function category, Binding and Catalytic Activity were the most prominently annotated terms, with significantly higher representation than other functional categories. These findings suggest that the DEGs are predominantly involved in cellular metabolic processes, responses to stimuli, the regulation of biological processes, and binding/catalytic activities of various biological enzymes.

To further elucidate the biological functions of DEGs, KEGG pathway enrichment analysis was performed to identify significantly enriched metabolic and signaling pathways (Figure 4D–F). At 10 days post-anthesis, DEGs were significantly enriched in pathways including biosynthesis of secondary metabolites, plant–pathogen interaction, plant hormone signal transduction, and phenylpropanoid biosynthesis. At 20 days post-anthesis, the most enriched pathways included plant–pathogen interaction, spliceosome, protein processing in the endoplasmic reticulum, ABC transporters, and circadian rhythm–plant. At 30 days post-anthesis, DEGs were predominantly enriched in metabolic pathways, biosynthesis of secondary metabolites, phenylpropanoid biosynthesis, glycolysis/gluconeogenesis, ABC transporters, flavonoid biosynthesis, and carotenoid biosynthesis. Notably, DEGs across all three developmental stages were significantly enriched in diterpenoid biosynthesis, isoflavonoid biosynthesis, zeatin biosynthesis, and anthocyanin biosynthesis pathways. Additionally, DEGs were enriched in porphyrin and chlorophyll metabolism as well as photosynthesis–antenna proteins pathways.

In conclusion, the formation of fruit color during green ripening in chili peppers is regulated by key metabolic and signaling pathways. Specifically, pigment metabolism involves phenylpropanoid biosynthesis (ko00940), flavonoid biosynthesis (ko00941), carotenoid biosynthesis (ko00906), isoflavonoid biosynthesis (ko00943), anthocyanin biosynthesis (ko00942), and porphyrin and chlorophyll metabolism (ko00860). Additionally, signaling pathways such as plant hormone signal transduction (ko04075), circadian rhythm–plant (ko04712), and photosynthesis–antenna proteins (ko00196) play critical roles in this process.

### 2.3. Chlorophyll Metabolism

**Figure 5:**
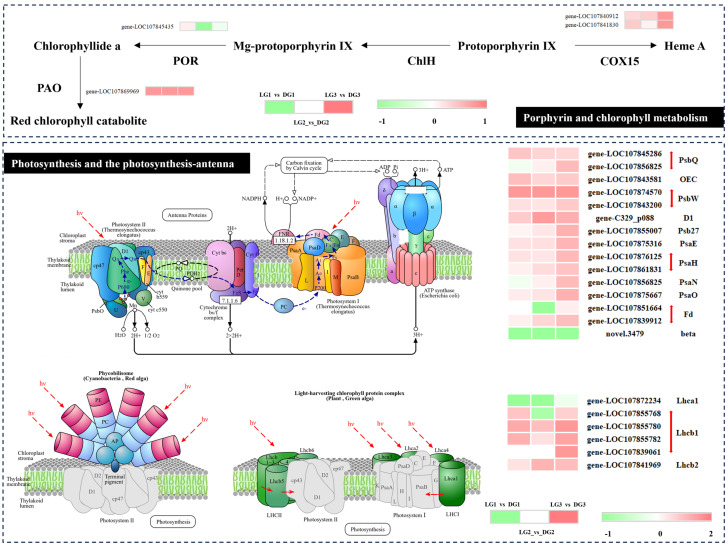
Heatmap representation of DEGs based on log_2_ fold change (log_2_FC). Green and red colors denote down-regulation and up-regulation of gene expression, respectively.

We analyzed DEGs associated with porphyrin and chlorophyll metabolism, photosynthesis, and photosynthesis–antenna proteins pathways (Figure 5). The analysis revealed that 4 DEGs were annotated to porphyrin and chlorophyll metabolism, 15 DEGs to the photosynthesis pathway, and 6 DEGs to the photosynthesis–antenna proteins pathway across three distinct stages of green ripening fruit color development. All genes exhibited up-regulation except for *novel.3479* and *gene-LOC107872234*, which showed down-regulation. These findings suggest that up-regulated genes play a critical role in the development of dark green fruit color in chili peppers.

### 2.4. Correlation Analysis of Flavonoid Metabolism

**Figure 6:**
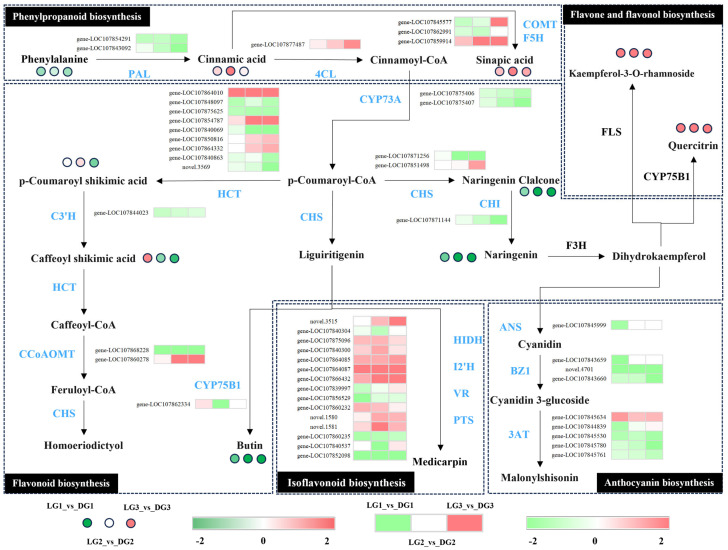
Differential gene and metabolite expression analysis. Changes in DEGs and DAMs are represented by log_2_FC. Green and red indicate the down-regulation and up-regulation of expression levels, respectively.

DAMs and DEGs were mapped to KEGG pathways to elucidate the relationship between key genes and metabolites associated with green ripe fruit color in pepper. All three stages showed significant enrichment in phenylpropanoid, flavonoid, and anthocyanin metabolism pathways, with flavonoids being the most abundant among differential metabolites. This enabled the construction of a gene-metabolite network (Figure 6). Across the three post-anthesis stages, we identified 47 DEGs and 10 DAMs involved in metabolic pathways regulating the biosynthesis of phenylpropanoids, flavonoids, anthocyanins, isoflavonoids, and flavonols.

In phenylpropanoid biosynthesis, two *PAL* genes (*gene-LOC107854291*, *gene-LOC107843092*) and one *F5H* gene (*gene-LOC107862991*) were down-regulated, while one *4CL* gene (*gene-LOC107877487*) and one *F5H* gene (*gene-LOC107859914*) were up-regulated.

In flavonoid biosynthesis, 11 genes were down-regulated, including 2 *CYP73A* genes (*gene-LOC107875406*, *gene-LOC107875407*), 5 *HCT* genes (*gene-LOC107848097*, *gene-LOC107875625*, *gene-LOC107840069*, *gene-LOC107840863*, *novel.3569*), 1 *C3′H* gene (*gene-LOC107844023*), 1 *CCoAOMT* gene (*gene-LOC107868228*), 1 *CHS* gene (*gene-LOC107871256*), and one *CHI* gene (*gene-LOC107871144*). Additionally, six genes were up-regulated, including four *HCT* genes (*gene-LOC107864010*, *gene-LOC107854787*, *gene-LOC107850816*, *gene-LOC107864332*), one *CCoAOMT* gene (*gene-LOC107860278*), and one *CHS* gene (*gene-LOC107851498*).

In isoflavonoid biosynthesis, four genes were down-regulated, including two *HIDH* genes (*gene-LOC107840304*, *gene-LOC107856529*), one *I2′H* gene (*gene-LOC107860235*), and one *PTS* gene (*gene-LOC107852098*). Nine genes were up-regulated, including six *HIDH* genes (*novel.3515*, *gene-LOC107875096*, *gene-LOC107840300*, *gene-LOC107864085*, *gene-LOC107864087*, *gene-LOC107866432*) and three *I2′H* genes (*gene-LOC107860232*, *novel.1580*, *novel.1581*).

In anthocyanin biosynthesis, two *BZ1* genes (*novel.4701*, *gene-LOC107843660*) and three *3AT* genes (*gene-LOC107845530*, *gene-LOC107845780*, *gene-LOC107845761*) were down-regulated, while one *3AT* gene (*gene-LOC107845634*) was up-regulated.

In the phenylpropanoid biosynthesis pathway, cinnamic acid (log_2_FC = 0.22 to 9.7) and sinapic acid (log_2_FC = 1.00 to 1.94) were up-regulated in DG, while phenylalanine was down-regulated (log_2_FC = −0.38 to −0.79). In the flavone and flavonol biosynthesis pathway, naringenin chalcone (log_2_FC = −0.89 to −4.27), naringenin (log_2_FC = −1.16 to −5.69), and butin (log_2_FC = −1.28 to −5.22) were down-regulated, while quercitrin (log_2_FC = 8.21 to 9.46) and kaempferol-3-O-rhamnoside (log_2_FC = 8.59 to 9.63) were up-regulated.

**Figure 7:**
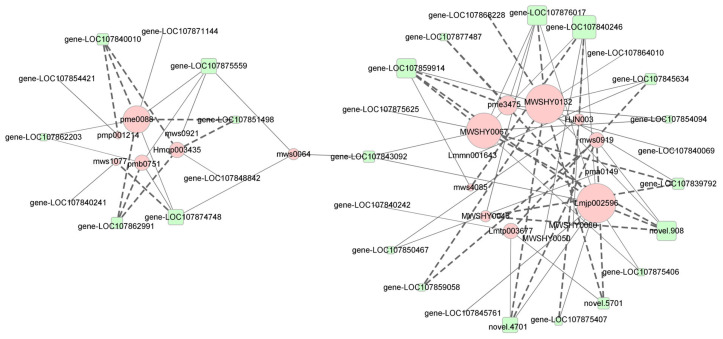
Network analysis of gene-metabolite correlations in flavonoid biosynthetic pathways. Green boxes represent genes, and red circles denote metabolites.

To further investigate the relationships between metabolites and genes in flavonoid biosynthetic pathways and identify Hub genes (highly connected genes), we calculated Pearson’s correlation coefficients between gene expression levels and metabolite abundances. Based on these correlation coefficients, a network diagram was constructed to visualize gene–metabolite interactions (Figure 7). Co-expression network analysis identified 20 DAMs and 31 DEGs, with four core phenylpropanoid biosynthetic genes—*scopoletin GTase* (*gene-LOC107840246*), *F5H* (*gene-LOC107859914*), *CCR* (*gene-LOC107876017*), and *CAD* (*novel.908*)—showing the highest connectivity to metabolite nodes. Notably, *scopoletin GTase* exhibited strong negative correlations with 1-O-sinapoyl-D-glucose (*r* = −0.88, *p* < 0.01) in phenylpropanoid biosynthesis (ko00940), while positively regulating quercetin-3-O-sambubioside (*r* = 0.86, *p* < 0.01) and rutin (*r* = 0.88, *p* < 0.01) in flavone and flavonol biosynthesis (ko00944). Conversely, *F5H* suppressed butin accumulation (*r* = −0.86, *p* < 0.01) in flavonoid biosynthesis and inhibited quercetin-3-O-sambubioside (*r* = −0.90, *p* < 0.01) and rutin (*r* = −0.86, *p* < 0.01) in ko00944. Intriguingly, *CCR* showed an inverse regulatory pattern to *F5H*, with significant positive correlations with butin (*r* = 0.83), quercetin-3-O-sambubioside (*r* = 0.87), and rutin (*r* = 0.83) (all *p* < 0.01). Furthermore, *CAD* mediated differential regulation in flavone and flavonol biosynthesis, positively correlating with quercitrin (*r* = 0.85, *p* < 0.01) and kaempferol-3-O-rhamnoside (*r* = 0.86, *p* < 0.01), while negatively correlating with rutin (*r* = −0.85, *p* < 0.01).

### 2.5. qRT-PCR Analysis

**Figure 8:**
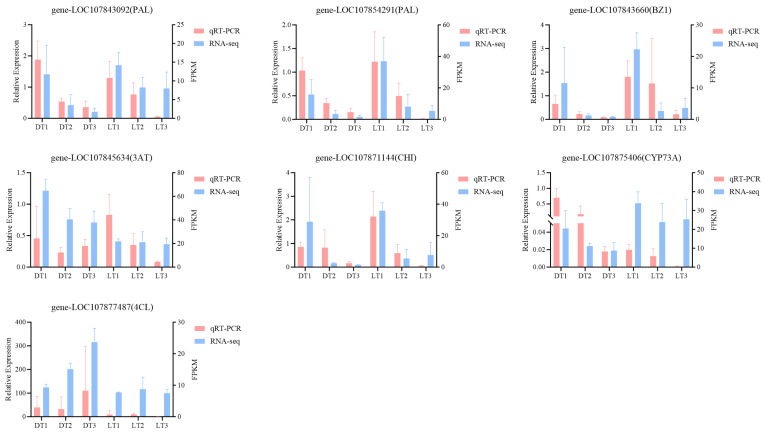
Validation of transcriptomic findings by qRT-PCR analysis. Quantitative data are presented as mean ± standard deviation (SD) from three independent biological replicates, with error bars representing SD.

To validate the reliability of RNA sequencing, seven candidate differentially expressed genes (DEGs) associated with chlorophyll metabolism and flavonoid biosynthesis pathways were selected for quantitative real-time PCR (qRT-PCR) analysis (Figure 8). The qRT-PCR results show high consistency with the gene expression patterns derived from transcriptome data. These findings confirm the reliability of the transcriptome sequencing data in this study.

## 3. Discussion

Fruit color is a key commercial trait in pepper, significantly influencing its market value. Capsicum fruits exhibit a diverse color palette, ranging from white, green, purple, and black at the immature stage to yellow, orange, red, and brown as the fruit matures [34,35]. Previous studies have demonstrated that chlorophyll, carotenoids, and flavonoids are the primary metabolites responsible for plant coloration. In this study, we identified candidate genes influencing green ripe fruit color in chili peppers by integrating metabolomic and transcriptomic analyses. Metabolite analysis revealed that variations in flavonoid, flavonol, isoflavone, and anthocyanin contents within the pigment metabolism pathway are pivotal factors determining fruit color divergence. RNA-seq transcriptomic analysis demonstrated that differentially expressed genes (DEGs) associated with anthocyanin biosynthesis, isoflavone metabolism, chlorophyll biosynthesis, and photosynthesis potentially regulate the pigmentation of green mature fruits. Furthermore, integrated transcriptomic and metabolomic analyses were employed to systematically investigate the flavonoid metabolic pathway.

Chlorophyll is a crucial pigment that determines pericarp coloration in many fruits. Key genes involved in chlorophyll biosynthesis have been identified in both leaves and fruits [36,37,38]. In this study, DEGs in porphyrin and chlorophyll metabolism pathways were annotated to *COX15*, *POR*, and *PAO*, with two *COX15* genes and one *PAO* gene showing significant up-regulation. Matile et al. demonstrated that inhibiting *PAO* gene expression leads to the accumulation of demagnesium chlorophyll a and inhibits chlorophyll degradation [39]. Pružinská et al. found that *PAO* gene expression positively correlates with chlorophyll breakdown rates in senescing Arabidopsis thaliana leaves [40]. In this study, PAO gene expression was significantly higher in dark green chili peppers compared to light green ones, potentially enhancing chlorophyll degradation. The pigment–protein complexes in higher plant chloroplasts are highly conserved, with photosystem II (PSII) containing six major chlorophyll a/b-binding proteins (Lhcb1-6). These proteins noncovalently bind pigments, including chlorophyll a, chlorophyll b, and lutein, forming the complexes LHCIIb, CP29, CP26, and CP24 [41,42]. The major complex, LHCIIb, is composed of a trimeric mixture of Lhcb1-3 proteins, which bind 60% of PSII chlorophyll [43]. The overexpression of *CaLhcb1.7* and *CaLhca5.1* in Han [44] increased chlorophyll content, enhanced net photosynthetic rate, and improved overall photosynthesis. Additionally, *CaLhcb1.7* and *CaLhca5.1* reduced reactive oxygen species (ROS) accumulation and improved plant resistance to pathogens and salt stress. Furthermore, the expression levels of *Lhcb1* and *Lhcb2* genes, involved in photosynthetic pathways, photosynthetic antenna protein synthesis, and metabolism, were significantly higher in dark green peppers compared to light green peppers. This suggests enhanced photosynthesis in dark green peppers, potentially leading to increased chlorophyll accumulation and deeper fruit coloration.

Flavonoids represent the most abundant class of secondary metabolites in plants. They serve as primary pigments responsible for the coloration of flowers, fruits, and leaves, while also playing critical roles in plant growth, development, and environmental stress responses [30,45,46,47]. Flavonoids are classified into five major subclasses: anthocyanins, flavanones, flavanonols, flavonols, and flavanols [44]. Previous studies have demonstrated that flavonoid metabolic pathways regulate the coloration of citrus fruit rinds [48], pepper fruit [30], melon rinds [49], and cucumber fruit [50]. Numerous studies have reported a positive correlation between flavonoid content and chlorophyll levels. Flavonoids, especially flavonols, exhibit significant antioxidant activity by scavenging ROS, mitigating oxidative damage to chloroplasts, and reducing chlorophyll degradation [51,52,53,54]. In this study, cinnamic acid, a key metabolite of the phenylpropanoid pathway, was significantly up-regulated in DG compared to LG. Furthermore, its derivatives, erucic acid and caffeic acid, also showed higher expression levels in DG. The significant up-regulation of phenylpropanoid metabolism, an upstream pathway of flavonoid biosynthesis, enhanced the flux of metabolites into anthocyanin, flavonoid, and related metabolic pathways. Quercitrin and kaempferol-3-O-rhamnoside, key flavonoid and flavonol compounds, were significantly up-regulated (log_2_FC = 8.21 to 9.63) across the three post-anthesis stages. This up-regulation may enhance ROS scavenging and inhibit chlorophyll degradation, thereby contributing to the intensification of green ripe fruit color in peppers. The *BZ1* gene (*novel.4701*; *r* = −0.98, log_2_FC = −9.29 to −9.70) and *CCR* gene (*novel.5701*; *r* = −0.91, log_2_FC = −5.71 to −7.18), which showed the strongest negative correlations with quercitrin in the flavonoid metabolic pathway, were significantly down-regulated. The gene most strongly correlated with kaempferol-3-O-rhamnoside, *BZ1* (*novel.4701*; *r* = −0.90), was also significantly down-regulated, while the *CAD* gene (*novel.908*; *r* = 0.86, log_2_FC = 7.23) was significantly up-regulated. These three genes may play key regulatory roles in the flavonoid metabolic pathway.

## 4. Materials and Methods

### 4.1. Experimental Materials and Sampling

The experimental materials comprised two pepper (*Capsicum annuum* L.) cultivars: ‘XHB’ (designated DG, exhibiting dark green pericarp coloration at the green maturity stage) and ‘QL2017’ (designated LG, demonstrating light green pigmentation at the green maturity stage), provided by the Horticulture Institute of Qinghai Academy of Agricultural and Forestry Sciences (Xining, China). These accessions display marked divergence in chlorophyll-based pigmentation during the green maturity phase while maintaining comparable agronomic characteristics. Both genotypes have exhibited consistent phenotypic stability through multi-annual observational evaluations.

Cultivation trials were initiated in early April 2020 within a controlled solar-heated greenhouse facility at the aforementioned institute. Fruit tissues were systematically sampled at three developmental stages (10, 20, and 30 days post-anthesis) with three biological replicates per time point. All collected specimens were immediately flash-frozen in liquid nitrogen and subsequently stored at −80 °C in ultra-low-temperature cabinets for subsequent biochemical analyses.

### 4.2. Metabolome Analysis

Metabolite analyses were carried out using a broadly targeted metabolomic approach developed by Wuhan Metware Biotechnology Co., Ltd. (Wuhan, China) (http://www.metware.cn/ accessed on 15 December 2021). Sample preparation commenced with the lyophilization of biological materials using a vacuum freeze-dryer (Scientz-100F, Ningbo Scientz Biotechnology Co., Ltd., Ningbo, China). Homogenization was subsequently performed with a mixer mill (MM 400, Retsch GmbH, Haan, Germany) at 30 Hz for 90 s utilizing zirconia beads. Precisely 100 mg aliquots of lyophilized powder underwent phased extraction with 1.2 mL 70% methanol aqueous solution, involving six intermittent vortex cycles (30 s agitation at 30 min intervals). Post-extraction, the samples were maintained at 4 °C for 12 h equilibration prior to centrifugation (12,000× *g*, 10 min). The supernatant was subjected to filtration through a 0.22 μm microporous membrane (SCAA-104, ANPEL Laboratory Technologies (Shanghai) Inc., Shanghai, China) before chromatographic separation on a UPLC-ESI-MS/MS system (ACQUITY UPLC HSS T3 column, 1.8 μm, 2.1 × 100 mm). Mobile phase constituents included solvent A (0.1% formic acid aqueous solution) and solvent B (0.1% formic acid acetonitrile) with the following gradient program: 0–1 min 5% B; 1–8 min 5–95% B; 8–9 min 95% B; 9–9.1 min 95–5% B; 9.1–11 min 5% B. Mass spectrometric detection was implemented via a Q TRAP^®^ 4500 system (AB Sciex LLC, Framingham, MA, USA) equipped with ESI source, operating in both positive/negative ionization modes. Instrument calibration involved serial injections of 10–100 μM polypropylene glycol solutions under standardized parameters: ion source temperature 550 °C, ion spray voltage ±5500 V, and curtain gas 35 psi. Differential metabolites were identified through orthogonal partial least squares–discriminant analysis (OPLS-DA) with the threshold criteria of variable importance in projection (VIP) ≥ 1.0 and absolute log_2_ fold change (|log_2_FC|) ≥ 1.0. Multivariate data pretreatment included log_2_ transformation and mean-centering, with permutation testing (n = 200 iterations) validating model robustness. Metabolite annotation was performed against the KEGG Compound Database (Release 107.0), followed by pathway enrichment analysis via metabolite set enrichment analysis (MSEA). The statistical significance of enriched pathways was determined through hypergeometric testing (*p* < 0.05).

### 4.3. Transcriptome Results Analysis

Total RNA was isolated from pepper fruit tissues using the RNAprep Pure Plant Kit (Tiangen Biotech, Beijing, China), with RNA integrity validated by Agilent 2100 Bioanalyzer (Agilent Technologies, Inc., Santa Clara, CA, USA) (RIN ≥ 7.0). Sequencing libraries were constructed from 1 µg high-quality RNA per biological replicate using the NEBNext^®^ UltraTM RNA Library Prep Kit for Illumina^®^ (New England Biolabs, Ipswich, MA, USA), following the manufacturer’s protocols with unique dual-index adapters for sample multiplexing. Raw sequencing data were filtered using fastp (v0.19.3) with (1) adapter trimming, (2) removal of reads containing >10% ambiguous bases (N), and (3) exclusion of reads with >50% low-quality bases (Q ≤ 20). Clean reads were aligned to the *Capsicum annuum* reference genome (https://www.ncbi.nlm.nih.gov/bioproject/PRJNA376668, accessed on 13 December 2021) using HISAT2 (v2.2.0) with default parameters. Gene expression quantification was performed via featureCounts (v1.6.2) and normalized as fragments per kilobase of transcript per million mapped reads (FPKM). Differentially expressed genes (DEGs) were identified using DESeq2 (v1.22.1) with thresholds of |log_2_(fold change)| ≥ 1.0 and Benjamini–Hochberg adjusted *p*-value (FDR) < 0.05. Functional enrichment analysis was conducted through clusterProfiler (v4.0.5) employing hypergeometric testing.

KEGG pathways: Kyoto Encyclopedia of Genes and Genomes database (Release 107.0).

GO terms: Gene Ontology Consortium annotations (15 January 2023.release).

Statistical significance was defined as *p*-value < 0.05 after false discovery rate correction.

### 4.4. Relevance Analysis

To elucidate the intricate relationship between genes and metabolites, differentially expressed genes (DEGs) and differentially accumulated metabolites (DAMs) within the same comparison group were annotated and mapped to the KEGG pathway. Pearson correlation analysis was conducted utilizing the quantitative expression profiles of genes and metabolites across all samples. The Pearson correlation coefficients were computed employing the ‘cor-function’ method implemented in R software(v3.5.1). Statistical significance was determined by applying stringent filtering criteria, with correlation coefficient threshold > 0.8 and *p*-value < 0.05. In the network graph, different node shapes represent genes or metabolites, with node size proportional to the degree of connectivity within the network. Edges between nodes indicate potential regulatory relationships, with solid lines representing positive correlations, dashed lines indicating negative correlations, and line thickness reflecting the strength of the correlation.

### 4.5. qRT-PCR Analysis

To validate the transcriptome sequencing results, seven differentially expressed genes (DEGs) were selected for quantitative real-time PCR (qRT-PCR) validation based on their functional annotations and expression patterns. Gene-specific primers were designed using Primer Quest Tool (Integrated DNA Technologies, Coralville, IA, USA). Total RNA was reverse-transcribed into cDNA using the Hifair^®^ III 1st Strand cDNA Synthesis SuperMix (Yeasen Biotechnology, Shanghai, China), following the manufacturer’s protocol to ensure complete genomic DNA removal. Quantitative PCR amplification was performed using Hieff^®^ qPCR SYBR Green Master Mix (Low Rox Plus) (Yeasen Biotechnology) on an Applied Biosystems 7500 Real-Time PCR System (Thermo Fisher Scientific, Waltham, MA, USA), with primer sequences provided in Supplementary Table S1. Each reaction was performed in triplicate using three independent biological replicates, and relative gene expression levels were calculated using the 2^−ΔΔCt^ method [55] with statistical analysis performed using GraphPad Prism 8.0 (GraphPad Software, San Diego, CA, USA).

## 5. Conclusions

This study suggests that the dark green pigmentation in pepper fruits arises from the synergistic interaction between flavonoid-mediated antioxidant activity and chlorophyll retention dynamics. Multi-omics integration revealed that elevated quercitrin and kaempferol-3-O-rhamnoside accumulation in XHB cultivars correlates with delayed chlorophyll degradation, potentially through ROS scavenging mechanisms. The transcriptional regulation of chlorophyll biosynthesis genes (*COX15*, *POR*) and light-harvesting components (*Lhcb1/2*) coincided with sustained chlorophyll levels, while the paradoxical upregulation of *PAO* suggests complex post-transcriptional control of catabolism. Co-expression networks identified *scopoletin GTase*, *F5H*, *CCR*, and *CAD* as central regulators of flavonoid flux, providing molecular targets for color modulation. Key limitations include the observational nature of metabolite–gene correlations and the absence of functional validation through transgenic approaches. Future studies should incorporate additional varieties to validate these findings on a broader scale, prioritizing the spatial–temporal resolution of chlorophyll turnover dynamics across diverse genetic backgrounds, coupled with metabolic flux analysis to quantify pathway contributions.

## Data Availability

The original contributions presented in the study are included in the article; further inquiries can be directed to the corresponding authors.

## Supplementary Materials

The following supporting information can be downloaded at: https://www.mdpi.com/article/10.3390/ijms26104508/s1.
