# Supplementary material for: Metabolic Pathways and Molecular Regulatory Mechanisms of Fruit Color Change During Greening Stage of Peppers (Capsicum annuum L.)
Source: Int J Mol Sci. 2025 May 9;26(10):4508. doi: 10.3390/ijms26104508 (PMC12111481; doi:10.3390/ijms26104508)
Supplement: Supplementary file 1 [file ijms-26-04508-s001.zip › ijms-3581289-supplementary.pdf]

Supplementary Materials

Supplementary Table s1. The table of qRT-PCR primer sequences.

Gene name	Primer	Sequence (5′-3′)
QRT-UBI	Forward primer	TGTCCATCTGCTCTCTGTTG
	Reverse primer	CACCCCAAGCACAATAAGAC
gene-LOC107843092	Forward primer	CTTGTCCCGTTGTCCTACATT
	Reverse primer	GGAACGCTTCCTCTGCATTA
gene-LOC107854291	Forward primer	CCCGAGTTCACTGACCATTT
	Reverse primer	CCTTCACATAAGAGCTACCATCC
gene-LOC107843660	Forward primer	ACTCCGTTCTGGAAAGCATATC
	Reverse primer	CCAAACACTCTCCACCAATCT
gene-LOC107845634	Forward primer	GTTGGACATGACGTTGAGAAGA
	Reverse primer	TGGAGTCCCTGGGTGAATATAG
gene-LOC107871144	Forward primer	ATCGTTCGCGATAGAAGGTAAG
	Reverse primer	ATTGTTGGCCAAGGAAGAAATAG
gene-LOC107875406	Forward primer	GAGGTTCTTGGAAGAGGAGAAG
	Reverse primer	ATTGGCAGCGCTAGGATAAT
gene-LOC107877487	Forward primer	GCTGTTCCGAGGAGTGATATG
	Reverse primer	GTTATGATGAGCTTCGCGTTTG

Supplementary Table s2. The table of transcriptome sequencing data statistics.

Sample	Raw Reads	Clean Reads	Clean Base (G)	Error Rate (%)	Q20 (%)	Q30 (%)	GC Content (%)
DG1-1	51115166	48509234	7.28	0.03	97.58	93.06	41.69
DG1-2	49636470	47436134	7.12	0.03	97.68	93.31	41.81
DG1-3	47348334	45157010	6.77	0.03	97.44	92.79	41.66
DG2-1	51603288	49084210	7.36	0.03	97.71	93.36	41.68
DG2-2	47020770	44877868	6.73	0.03	97.41	92.71	41.7
DG2-3	47899392	45976918	6.9	0.03	97.65	93.28	41.74
DG3-1	48923972	46075334	6.91	0.03	97.56	93.08	41.94
DG3-2	52505838	50664736	7.6	0.03	97.25	92.17	42.46
DG3-3	47616478	46088842	6.91	0.03	97.56	93.08	42.16
LG1-1	48115416	46430814	6.96	0.03	97.48	92.92	41.93
LG1-2	49295772	47404852	7.11	0.03	97.34	92.59	41.88
LG1-3	47393024	45229698	6.78	0.03	97.52	93	42.3
LG2-1	48000250	45914194	6.89	0.03	97.46	92.89	42.03
LG2-2	50396836	48123038	7.22	0.03	97.56	93.05	42.18
LG2-3	48872524	46061018	6.91	0.03	97.56	93.11	42.14
LG3-1	47375452	44663340	6.7	0.03	97.61	93.26	41.96
LG3-2	45103900	42459060	6.37	0.03	97.9	93.83	42.12
LG3-3	48862064	45702206	6.86	0.03	97.46	92.94	42.29
